# Revisiting the segmentation threshold for Lu‐177 SPECT

**DOI:** 10.1002/mp.70399

**Published:** 2026-03-19

**Authors:** Yibin Liu, Jian Yang, Peng Wang, Yingwei Wang, Yue Chen, Greta S. P. Mok

**Affiliations:** ^1^ Biomedical Imaging Laboratory (BIG), Department of Electrical and Computer Engineering, Faculty of Science and Technology University of Macau Taipa Macau SAR China; ^2^ Department of Nuclear Medicine Affiliated Hospital of Southwest Medical University Luzhou Sichuan China; ^3^ Ministry of Education Frontiers Science Center for Precision Oncology Faculty of Health Science, University of Macau Taipa Macau SAR China

**Keywords:** ^177^Lu, SPECT/CT, threshold, tumor segmentation

## Abstract

**Background:**

The mostly used threshold‐based segmentation method for SPECT, i.e., 42% of the maximum intensity, was derived from ^99m^Tc and may not be directly applicable to ^177^Lu.

**Purpose:**

This study aims to revisit the optimal segmentation threshold for ^177^Lu SPECT.

**Methods:**

A cylindrical Jaszczak phantom containing six spheres (2–113 mL) was imaged via simulation and physical experiments using a clinical dual‐head NaI SPECT/CT system. The spheres were filled with ^99m^Tc and ^177^Lu, with different sphere‐to‐background ratios (SBRs). One hundred and twenty projections were acquired and reconstructed using filtered back‐projection (FBP) and 3D ordered subset expectation maximization (OS‐EM) algorithms with attenuation and scatter corrections, followed by Gaussian filtering (*σ* = 3.8 mm). Thresholds from 1% to 99% (1% interval) of peak intensity were applied to minimize the absolute volume error (AVE) of the spheres. The newly derived ^177^Lu threshold was further validated on ^177^Lu‐PSMA‐617 (*n* = 6), ^177^Lu‐DOTATATE (*n* = 5), ^177^Lu‐FAP‐2286 (*n* = 5) and ^177^Lu‐DOTA‐IBA (*n* = 4) SPECT images, comprising 45 tumors with manual segmentations used as reference. Mean Dice, HD95, and AVE were calculated for all tumors and compared between the conventional threshold (42%) and the newly derived threshold using the Mann–Whitney *U* test.

**Results:**

The optimal threshold increased along with the decrease in sphere volume or SBR. For SBR ≥ 3.5:1 and volume ≥ 16 mL, the mean optimal threshold of ^177^Lu converged to 56% for FBP and 50% for OS‐EM. The derived 50% threshold significantly improved tumor segmentation performance compared to the 42% threshold, with a higher Dice score (0.5999 ± 0.1589 vs. 0.6694 ± 0.1361) (*p *< 0.05), lower HD95 (2.0070 ± 1.1508 mm vs. 1.7392 ± 1.0643 mm), and lower AVE (130.72% ± 101.87% vs. 69.21% ± 63.49%) (*p *< 0.05).

**Conclusions:**

An optimal ^177^Lu‐specific threshold (∼50%) was derived and clinically validated, differing from the conventional 42% threshold used for ^99m^Tc. The new threshold improved segmentation accuracy across different therapeutic radiopharmaceutical distributions.

**Abbreviations**
AC: Attenuation correction
AI: Artificial intelligence
AVE: Absolute volume error
CT: Computed Tomography
DOTA‐IBA: DOTA‐Ibandronate
DOTATATE: DOTA‐[Tyr^3^]‐octreotate
DOTATOC: DOTA‐[Tyr^3^]‐octreotide
FAPI: Fibroblast activation protein inhibitors
FBP: Filtered back‐projection
HD95: Hausdorff Distance at 95th percentile
OS‐EM: Ordered subset expectation maximization
PSMA: Prostate specific membrane antigen
SBR: Sphere‐to‐background ratio
SC: Scatter correction
SPECT: Single‐Photon Emission Computed Tomography
VOI: Volume‐of‐interest

## 1 INTRODUCTION

Radiopharmaceutical therapy^1^ is recognized as an effective means for treating a variety of cancers with ^177^Lu‐labeled radiopharmaceuticals, such as ^177^Lu‐prostate specific membrane antigen (PSMA) for prostate cancers,^2^ ^177^Lu‐fibroblast activation protein inhibitors (FAPI) for solid tumors,^3^ ^177^Lu‐DOTA‐[Tyr^3^]‐octreotate (DOTATATE)^4^/DOTA‐[Tyr^3^]‐octreotide (DOTATOC) for neuroendocrine tumors^4^, ^5^ and ^177^Lu‐DOTA‐Ibandronate (DOTA‐IBA) for metastatic bone tumors.^6^ Personalized treatment planning ensures that the tumor reaches the required absorbed dose threshold while maintaining the absorbed dose of critical organs in safety levels. Reliable dosimetry requires robust and accurate segmentation methods.

Thresholding is a widely used semi‐automatic segmentation method in Single‐Photon Emission Computed Tomography (SPECT) imaging. Commonly, a fixed threshold of 42% of the maximum voxel intensity within the volume‐of‐interest (VOI) is employed.^7^ The optimal threshold is influenced by several factors, including the VOI‐to‐background ratio, the size and shape of the VOI, as well as image noise and resolution.^8^ In particular, for small volumes, partial volume effects are more pronounced, leading to increased boundary blurring.^9^, ^10^ The 42% threshold was derived decades ago from ^99m^Tc SPECT images reconstructed using filtered back‐projection (FBP) and attenuation correction (AC) with Chang's method. The energy spectrum of ^99m^Tc differs that of^177^Lu significantly, e.g., photopeak in 140 keV versus 113 and 208 keV. Therefore, medium‐energy collimators are recommended for ^177^Lu while low‐energy high‐resolution collimators are used for ^99m^Tc. Besides, modern hybrid SPECT/Computed Tomography (CT) systems incorporate advanced reconstruction techniques, including ordered subset expectation maximization (OS‐EM), CT‐based AC. Therefore, the 42% threshold may not be suitable for accurate VOI segmentation in recent ^177^Lu SPECT imaging. This study aims to revisit the optimal segmentation threshold for ^177^Lu SPECT by systematically evaluating both phantom and clinical data.

## 2 METHODS AND MATERIALS

### 2.1 Simulation study

#### 2.1.1 Digital phantom

**FIGURE 1:**
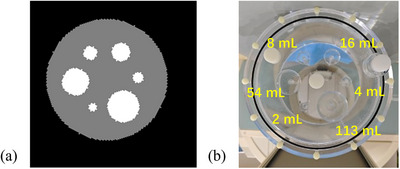
(a) Sample axial slices of the digital geometrical phantom. (b) The Jaszczak phantom with spheres inserted.

A computer‐based digital cylindrical phantom was designed based on a standard Jaszczak phantom, with a diameter of 22.2 cm and a length of 19.5 cm. The phantom simulated a water‐equivalent background and contained spherical regions with volumes of 2, 4, 8, 16, 54 and 113 mL. All spheres were positioned on the central axial plane and arranged equidistantly on a concentric circle with a radius of 5.5 cm (Figure 1a). The spheres were simulated with a uniform activity concentration of 296 kBq/mL for ^99m^Tc and 1628 kBq/mL for ^177^Lu separately. The sphere‐to‐background ratios (SBRs) were set to be no background, 10:1, 5:1, 3.5:1 and 2:1 respectively. The matrix size and voxel size of the phantom were 256 × 256 × 256 and 2.4 × 2.4 × 2.4 mm^3^, respectively.

#### 2.1.2 Monte Carlo simulation

The SIMIND Monte Carlo program v.7.0.3^11^ was used to simulate a clinical dual head NaI(Tl) SPECT/CT system (Symbia T16, Siemens Healthiness, Germany) with low‐energy high‐resolution parallel hole collimators for ^99m^Tc imaging and medium‐energy general‐purpose parallel hole collimators for ^177^Lu imaging, using the digital phantom and its corresponding attenuation maps at 140 keV (^99m^Tc) and 208 keV (^177^Lu) as input. One hundred and twenty noise‐free projections with a fixed radius‐of‐rotation of 24 cm were generated over 360° at a single bed position for the geometrical phantom, modeling attenuation, scatter, interactions within collimator‐detector and back‐scatter. For ^99m^Tc imaging, the primary energy window was centered at 140 keV with a 15% window width (129.3–150.3 keV). A scatter window was set at 108.4–129.3 keV for the dual energy window scatter correction (SC). For ^177^Lu imaging, the primary energy window centered at 208 keV with a 15% window width (192.4–223.6 keV). Two scatter windows were set at 161.2–192.4 keV and 223.6–244.4 keV for the triple energy window SC. Realistic noisy projections were generated by applying Poisson noise to scaled noise‐free projections, with total counts set to 3.8 × 10^6^ (no background), 1.9 × 10^7^ (SBR 10:1), 3.0 × 10^7^ (SBR 5:1), 4.1 × 10^7^ (SBR 3.5:1), and 7.1 × 10^7^ (SBR 2:1) for ^9^ ^9^ ^m^Tc, and 7.5 × 10^6^, 3.3 × 10^7^, 3.4 × 10^7^, 4.7 × 10^7^, and 7.8 × 10^7^, respectively, for ^1^ ^7^ ^7^Lu. To model the effect of continuous‐to‐discrete activity sampling of the activity distribution as seen in the clinical data acquisition,^12^ the matrix size for projections was collapsed from 256 × 256 with a bin size of 2.4 × 2.4 mm^2^ to 128 × 128 with a bin size of 4.8 × 4.8 mm^2^.

### 2.2 Physical phantom study

#### 2.2.1 Physical phantom

A cylindrical Jaszczak phantom (Data Spectrum Corporation, NC, USA) with a diameter of 22.2 cm and a height of 19.5 cm, consistent with the digital phantom, was used. Six spheres, with volumes identical to those in the digital phantom (2, 4, 8, 16, 54, and 113 mL), were filled with water solutions containing 296 kBq/mL of ^99m^Tc and 1628 kBq/mL of ^177^Lu respectively. The activity of the ^99m^Tc and ^177^Lu solutions was measured using a dose calibrator (CRC‐55tR, Capintec, Florham Park, NJ, USA). Based on the manufacturer's specifications (accuracy better than ± 2%), the activity measurement uncertainty was assumed to be approximately ± 2%.^13^ All spheres were arranged equidistantly along a concentric circle with a radius of 5.5 cm (Figure 1b) and placed inside the Jaszczak phantom. Initially, the phantom was filled with water to simulate the no background (i.e., background activity = 0). Subsequently, background activity was added to achieve SBRs of 10:1, 5:1, 3.5:1, and 2:1.

#### 2.2.2 Data acquisitions

SPECT scans were performed using a conventional dual‐head NaI(Tl) SPECT system (Symbia T16, Siemens Healthiness, Germany). The phantom was positioned at the center of the field of view. A total of 120 projections were acquired over 360° at a fixed radius‐of‐rotation of 24 cm, using an acquisition time of 20–30 s per view for ^99m^Tc imaging based on the decaying activity to ensure consistent image quality across scans and 25 s per view for ^177^Lu imaging. The scatter energy windows were set consistent with those used in the simulation study. Each projection had a matrix size of 128 × 128 with a pixel size of 4.8 × 4.8 mm^2^. Corresponding CT scans (130 kV, 190 mA, pitch: 0.8) were acquired over the same region and served as AC maps, with a voxel size of 0.98 × 0.98 × 2 mm^3^ (512 × 512 × 226).

#### 2.2.3 Image reconstruction

Both simulated and real projections of ^99m^Tc and ^177^Lu were reconstructed using (1) FBP with a Butterworth filter (cutoff frequency: 0.4, order 8 for ^99m^Tc; cutoff frequency: 0.4, order 5 for ^177^Lu), SC and Chang's AC; (2) 3D OS‐EM (4 iterations, 8 subsets), SC and CT‐based AC. The attenuation maps from the phantoms were used for AC in the phantom study. The reconstruction parameters were selected according to the vendor‐recommended protocol. The reconstruction matrix size and voxel size of SPECT images were 128 × 128 × 128 and 4.8 × 4.8 × 4.8 mm^3^, respectively. A post‐reconstruction Gaussian filter (*σ* = 3.8 mm) was applied on the reconstructed images.

### 2.3 Clinical study

SPECT/CT images from 20 patients underwent treatment with ^177^Lu‐PSMA‐617 (*n* = 6), ^177^Lu‐DOTATATE (*n* = 5), ^177^Lu‐FAP‐2286 (*n* = 5) and ^177^Lu‐DOTA‐IBA (*n* = 4) were analyzed retrospectively under the local ethics approval from the Affiliated Hospital of Southwest Medical University. Ninety projections over 360̊ with 15 s per projection were obtained for 1–2 bed positions, using a conventional dual‐head NaI(Tl) SPECT system (Symbia T16, Siemens Healthiness, Germany) with medium‐energy general‐purpose collimators. The energy window settings were consistent with the phantom study. Corresponding CT (130 kV, 27–128 mA, pitch: 1.4) were acquired covering the same region and served as AC maps, with a voxel size of 0.98 × 0.98 × 5 mm^3^ (512 × 512 × varying length). Projections were reconstructed using 3D OS‐EM algorithm (4 iterations, 8 subsets), triple energy window SC, and CT‐based AC. The post‐reconstruction Gaussian filter (*σ* = 3.8 mm) was applied. The matrix size and voxel size of the SPECT reconstruction were 128 × 128 × varying length and 4.8 × 4.8 × 4.8 mm^3^. The SPECT/CT images were registered using the rigid plus B‐spline transformation under the open‐source program “Elastix”.^14^

### 2.4 Data analysis

A range of threshold values from 1% to 99% with 1% interval was evaluated in the phantom studies. The optimal thresholds of spheres of varying volumes and SBRs were identified as those that minimized the absolute volume error ($AVE_{sphere}$) between the segmented volume and the known volume of the sphere.

(1) $$\mathrm{AV}E_{\mathrm{sphere}}=\left|\frac{V_{\mathrm{Segmented}}-V_{\mathrm{Ground}\;\mathrm{truth}}}{V_{\mathrm{Ground}\;\mathrm{truth}}}\right|$$

where $V_{Segmented}$ is the volume of segmented binary mask for a specific segmenting threshold and a particular size of sphere, $V_{Ground\;truth}$ is the sphere volume obtained from manual CT‐based segmentation. Comparisons of optimal thresholds from different radionuclides (^99m^Tc vs. ^177^Lu) and reconstruction methods (FBP vs. OS‐EM) were performed using the Mann–Whitney *U* test.

For the clinical study, the segmentation results using the conventional thresholds (42%) and the newly derived threshold based on the phantom studies were compared using the Dice, Hausdorff Distance at 95th percentile (HD95) and mean AVE ($MAVE_{tumor})$ with the Mann–Whitney *U* test.

(2) $$\mathrm{MAV}E_{\mathrm{tumor}}=\frac{1}{n}\;\sum_{1}^{n}\left|\frac{V_{\mathrm{Segmented}\;\mathrm{tumor}}-V_{\mathrm{Ground}\;\mathrm{truth}\;\mathrm{tumor}}}{V_{\mathrm{Ground}\;\mathrm{truth}\;\mathrm{tumor}}}\right|$$

where $V_{Segmented\;tumor}$ is the volume of segmented binary mask using the 2 thresholds for tumor, $V_{Ground\;truth\;tumor\;}$ is the volume of tumor masks manually segmented from CT and *n* is the number of tumors. A total of 45 tumors from 20 patients were manually delineated through consensus by three nuclear medicine clinicians with five‐year experience using fused SPECT/CT images as the ground truth masks. Anatomical information provided by CT was used as a reference to guide the boundary definition of the tumors, if applicable, ensuring consistency and accuracy across different cases. Only tumors visible on CT would be analyzed.

## 3 RESULTS

### 3.1 Phantom study

**FIGURE 2:**
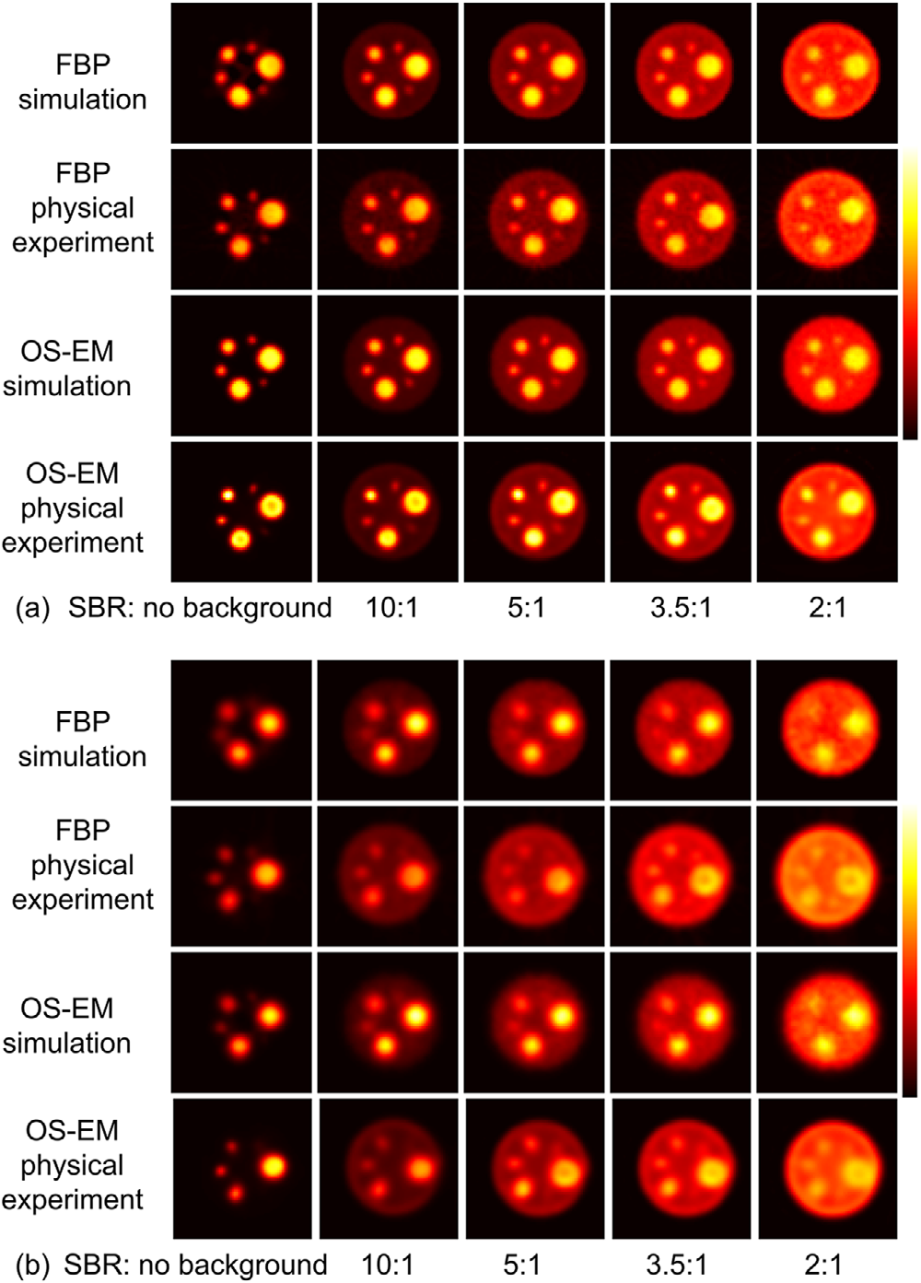
The reconstruction images of digital phantom and physical phantom for (a) ^99m^Tc SPECT and (b) ^177^Lu with FBP and OS‐EM.

**FIGURE 3:**
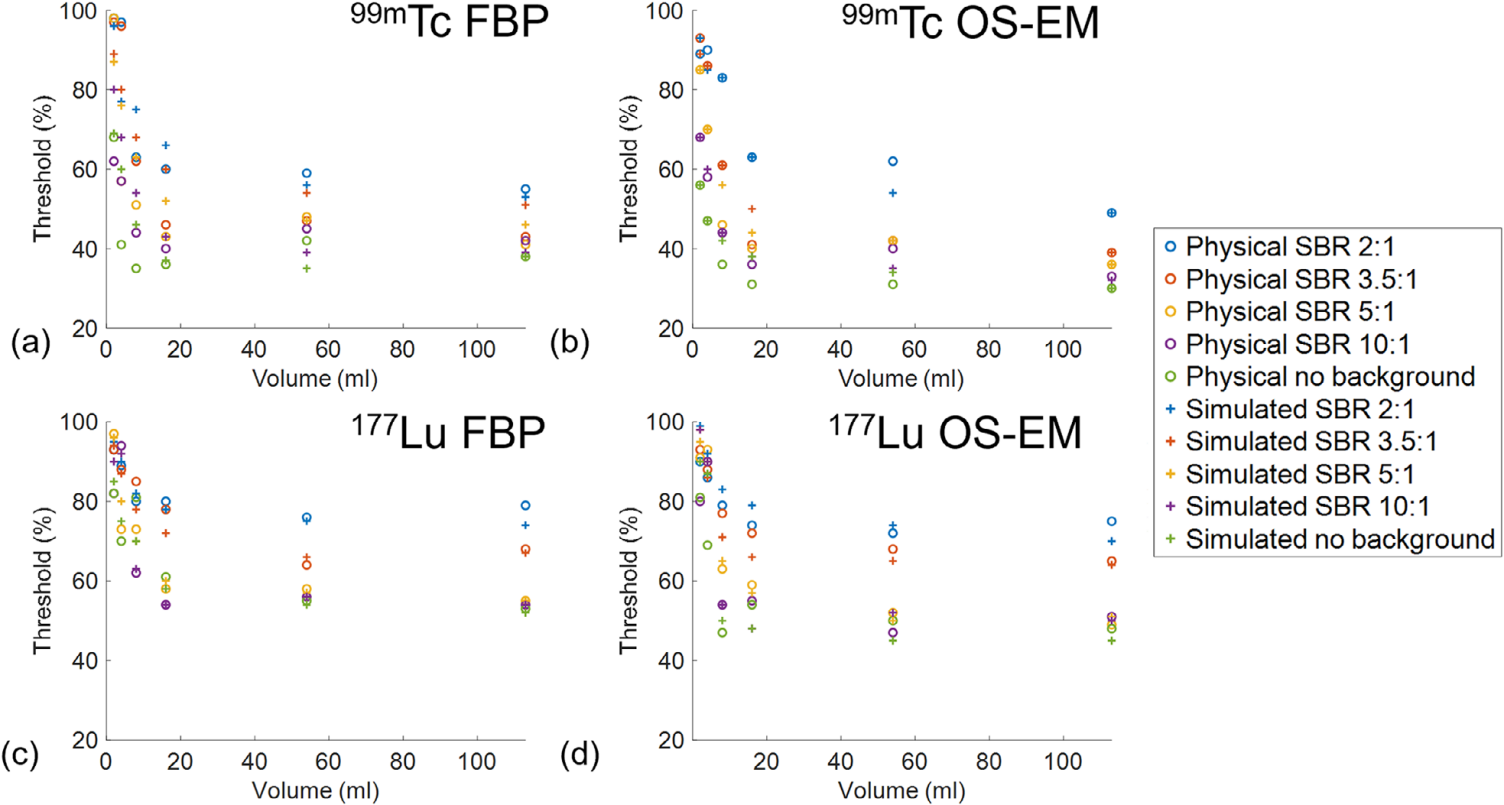
The optimal thresholds vary with the sphere volumes and SBRs for (a) ^99m^Tc SPECT and (b) ^177^Lu SPECT with FBP reconstruction and (c) ^99m^Tc SPECT and (d) ^177^Lu SPECT with OS‐EM reconstruction for simulation and physical experiments.

The reconstructed images of the digital phantom and physical phantom for ^99m^Tc and ^177^Lu are shown in Figure 2. The images from the simulation data and the physical experimental data show high visual similarity, indicating the accuracy of the Monte Carlo simulations. Compared to ^99m^Tc, the image quality of ^177^Lu reconstructions is generally lower, with reduced contrast. Figure 3 illustrates the relationship between optimal threshold values for different sphere volumes and SBRs for ^99m^Tc and ^177^Lu. The optimal threshold increased along with the decrease in sphere volume or SBR. Notably, for spheres with SBR > 3.5:1 and volumes ≥ 16 mL, the variation of optimal thresholds becomes less pronounced. Moreover, the thresholds derived from simulation data closely match those from physical experimental data across the range of volumes and SBRs evaluated, again confirming the validity of the simulation model.

The optimal thresholds for ^177^Lu were consistently higher than those of ^99m^Tc (*p *< 0.05) across spheres with SBR > 3.5:1 and volumes ≥ 16 mL. Specifically, the mean optimal threshold across these spheres for ^99m^Tc converges to approximately 43% for FBP, consistent with the previous reference,^7^ and 38% for OS‐EM reconstruction. For ^177^Lu, the mean optimal threshold across spheres with SBR > 3.5:1 and volumes ≥ 16 mL is higher, converging to 56% for FBP and 50% for OS‐EM. Furthermore, OS‐EM reconstruction yields significantly lower optimal thresholds compared to FBP for both isotopes (*p* < 0.05), reflecting the improved image quality associated with the iterative reconstruction method. The threshold for ^177^Lu was therefore recommended as 50% as most ^177^Lu reconstructed images are based on OS‐EM.

### 3.2 Clinical study

Using the new 50% threshold, the mean Dice coefficient is increased from 0.5999 ± 0.1589 to 0.6694 ± 0.1361 (*p* < 0.05). Although the mean HD95 value is decreased from 2.0070 ± 1.1508 mm to 1.7392 ± 1.0643 mm with the new threshold, the difference is not statistically significant (*p* > 0.05). In terms of $MAVE_{tumor}$ of the tumors, the 50% threshold substantially reduces the error from 130.72% ± 101.87% to 69.21% ± 63.49% (*p* < 0.05). Table 1 summarizes Dice results obtained using two segmentation thresholds, stratified by tumor volume.

**TABLE 1:** Dice, HD95 and volume error segmented from two thresholds in the clinical study, stratified by tumor volume.

Volume range (mL)		< 16	16–30	30–50	Total
Number of tumors		15	21	9	45
Dice	(50% threshold)	0.6127 ± 0.1261	0.6801 ± 0.1256	0.6884 ± 0.1423	0.6694 ± 0.1361
	(42% threshold)	0.5723 ± 0.1383	0.5796 ± 0.1586	0.6263 ± 0.1597	0.5999 ± 0.1589
HD95 (mm)	(50% threshold)	2.6137 ± 1.3121	1.6833 ± 1.0135	1.2929 ± 0.4353	1.7392 ± 1.0643
	(42% threshold)	4.0826 ± 3.9261	1.9221 ± 1.1016	1.5471 ± 0.5412	2.0070 ± 1.1508
$MAVE_{tumor}$	(50% threshold)	78.97% ± 76.83%	52.45% ± 77.36%	37.55% ± 88.95%	69.21% ± 63.49%
	(42% threshold)	160.86% ± 113.88%	123.37% ± 113.28%	100.31% ± 128.07%	130.72% ± 101.87%

**FIGURE 4:**
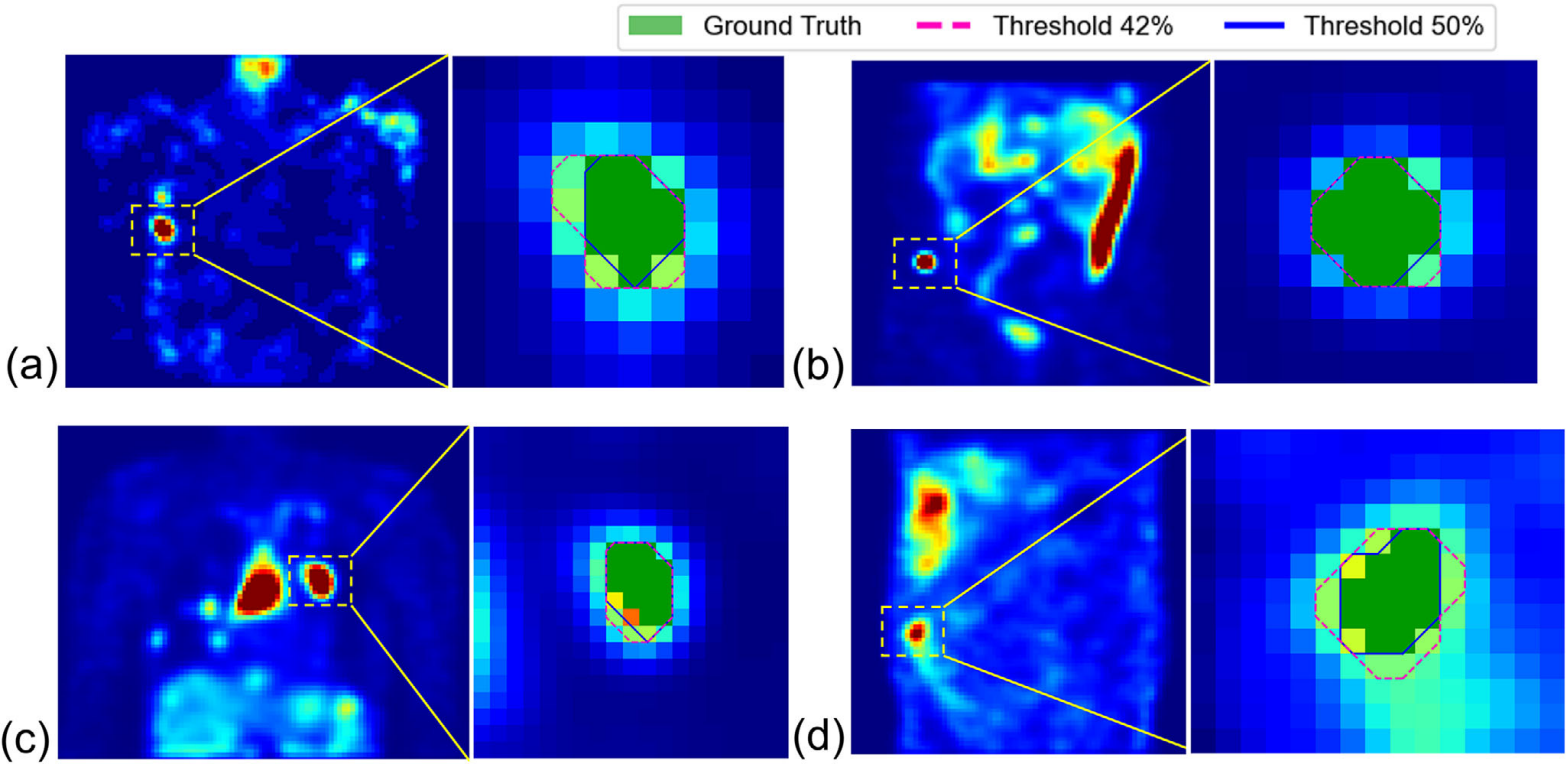
Comparison of segmentation masks from (a) ^177^Lu‐DOTA‐IBA, (b) ^177^Lu‐PSMA‐617, (c) ^177^Lu‐DOTATATE, and (d) ^177^Lu‐FAPI‐2286 using different new and conventional thresholding values.

Figure 4 provides a visual comparison of segmentation outcomes using two fixed thresholds (42% vs. 50%) across four representative ^1^ ^7^ ^7^Lu‐labeled radiopharmaceuticals. The magenta dashed lines (threshold 42%) typically show bigger coverage than the blue contours (threshold 50%), suggesting a tendency of over‐segmentation on small tumors.

## 4 DISCUSSION

In this study, for ^177^Lu SPECT, the optimal segmentation thresholds are consistently higher than those for ^99m^Tc, primarily due to its poorer spatial resolution. The OS‐EM reconstruction method significantly improves image quality, allowing for lower and more accurate threshold determination compared to FBP. The optimal threshold increased along with the decrease in sphere volume or SBR. This is mainly attributable to the partial volume effect, which is more pronounced for spheres < 20 mL and reduced with increasing volume. Besides, inferior spatial resolution and increased noise lead to blurred VOI boundaries and reduced tumor‐to‐background contrast. Therefore, a higher threshold is necessary to include the target tumor VOI. Conversely, higher quality images provide clearer tumor delineation and higher contrast, and lower thresholds are suitable to capture the true tumor extent.

When SBR > 3.5:1 and sphere volume ≥ 16 mL, the threshold values tend to converge, suggesting the potential for adopting fixed thresholds in clinical applications and a single threshold (50%) was derived by averaging these optimal thresholds. Overall, these findings underscore the importance of isotope‐ and reconstruction‐specific threshold optimization for accurate SPECT quantification. Our clinical study indicated the proposed 50% threshold in ^1^ ^7^ ^7^Lu imaging shows strong translational potential for routine clinical use, outperforming the conventionally used 42% threshold, especially in terms of Dice score and $MAVE_{tumor}$ across different therapeutic radiopharmaceuticals. Further stratified analysis revealed that segmentation performance based on the new threshold was better for tumors of different sizes as compared to the old threshold (Table 1).

Furthermore, this study compares the threshold‐based segmentation performance between simulation and physical phantom experiments for both ^99m^Tc and ^177^Lu SPECT images. The consistency observed between simulation and experimental results validates the simulation model. Simulations enable radiation‐free experiments with control over different acquisition parameters. This approach provides ultimate ground truth for evaluations and insights on threshold optimization prior to physical phantom experiments.

Recent advances in artificial intelligence (AI) have enabled adaptive and automatic segmentation approaches. Chen et al.^15^ proposed a convolutional neural network trained with fuzzy C‐means cluster‐based loss functions, enabling unsupervised, semi‐supervised, and supervised segmentation of bone and bone lesion on SPECT/CT images. The proposed semi‐supervised model achieves a Dice score of 0.75 for bone lesion segmentation, outperforming the supervised model which achieved a Dice score of 0.4. Chaichana et al.^16^ developed convolutional neural networks for automated segmentations of the lungs, liver, and hepatic tumors using the CT component of ^99m^Tc‐MAA SPECT/CT scans, achieving high segmentation accuracy. However, misalignment between SPECT and CT due to patient motion, respiratory artifacts, or acquisition mismatch could reduce spatial registration accuracy, affecting dosimetry.^17^ Contrast‐enhanced CT modalities like CT arterial portography and CT hepatic arteriography could improve lesion visibility and segmentation, especially for hepatocellular carcinoma.^18^ However, for ^177^Lu‐PSMA and ^177^Lu‐FAPI treatments, tumors may lack morphological distinctiveness on CT, making CT‐based segmentation less feasible. Robust AI segmentation models for whole‐body tumor delineation on ^177^Lu SPECT/CT images are warranted and should be further investigated. Besides, the generalizability and explainability of most of current AI‐based methods need to be further validated. Therefore, the fixed threshold‐based segmentation is still the clinical mainstream and worth pursuing for new theranostic tracers.

A limitation of this study is that the number of projections (*n* = 120) used in phantom experiments differed from that used in clinical acquisitions (*n* = 90), due to the routine clinical protocol in our center to reduce acquisition time. The impact of using different numbers of projections and reconstruction parameters (e.g., iteration numbers) on segmentation threshold is beyond the scope of this study but is expected to be insignificant relatively. Although optimal thresholds actually depend on lesion size and SBR, size‐ or SBR‐specific thresholds are difficult to implement in practice because lesion size and SBR are generally unknown before segmentation. This simplification may limit segmentation accuracy for very small or low‐contrast lesions thus results should be interpreted with special attention. The use of a single threshold, similar to previous studies,^7^, ^19^ represents a pragmatic compromise between segmentation accuracy and practical applicability.

## 5 CONCLUSION

An optimal ^177^Lu‐specific threshold (∼50%) was derived and clinically validated, differing from the conventional 42% threshold used for ^99m^Tc. The new threshold improved segmentation accuracy across different therapeutic radiopharmaceutical distributions.

## CONFLICT OF INTEREST STATEMENT

The authors declare no conflicts of interest.

## ETHICS APPROVAL

The patient data used in this study are under ethics from Affiliated Hospital of Southwest Medical University (21008, 2021LZXNYD).

## Data Availability

Authors will share data upon request to the corresponding author (Prof. Yue Chen).
